# Potential and limitations of a large language model in the statistical review of comparative categorical data: an exploratory study of structured prompt-guided approach

**DOI:** 10.1186/s41073-026-00231-0

**Published:** 2026-07-01

**Authors:** Min Dong, Xuemei Liu, Yunmei Luo, Yuanxiang Zeng, Fang Fei, Liang Du

**Affiliations:** 1 West China Medical Publisher, West China School of Medicine (West China Hospital), Sichuan University, Chengdu, 610041 P. R. China; 2 Center for Education of Medical Humanities, West China School of Medicine (West China Hospital), Sichuan University, Chengdu, 610041 P. R. China; 3 School of Literature and Journalism (School of Publishing) of Sichuan University, Chengdu, 610207 P. R. China; 4 Chinese Evidence-based Medicine Center, West China School of Medicine (West China Hospital), Sichuan University, Chengdu, 610041 P. R. China; 5 Innovation Institute for Integration of Medicine and Engineering, West China School of Medicine (West China Hospital), Sichuan University, Chengdu, 610041 P. R. China

**Keywords:** Large Language Model, Statistical Review, Structured Prompt, Prompt Engineering, Human-Computer Collaboration, Research Integrity, Accountability

## Abstract

**Background:**

Statistical review is essential for research quality and integrity, yet traditional manual review is inefficient. Large language models (LLMs) offer potential support but are unreliable when used without guidance for precise calculations and raise concerns about accountability. This study evaluated whether a structured, rule-based prompt can reliably constrain an LLM to perform statistical review of comparative categorical data, and characterized both its feasibility and its inherent risks from an accountability perspective.

**Methods:**

This study employed a two-stage design based on the DeepSeekV3.2. In the first stage, a structured prompt was developed through dozens of "test-fail-iterate" cycles using 20 published medical articles. The prompt assigned the LLM the role of a "statistics expert" and provided a closed set of computational rules and a "recognize data-select calculation formula-calculate" workflow for analyzing categorical data, including Pearson's Chi-square test, continuity correction, and McNemar's tests. In the second stage, the performance of the final prompt was evaluated on a test set of 20 independent manuscripts. The model's output was compared against the results calculated by a senior statistician (the gold standard). The primary outcome measures were the performance in statistical method selection and numerical computation, including accuracy, sensitivity (recall), specificity, positive predictive value, negative predictive value, F1 score, and Cohen's Kappa. Secondary measures included reproducibility and efficiency.

**Results:**

The test set consisted of 15 manuscripts with independent samples and 5 with paired samples. In the assessment of the appropriateness of statistical method selection for 148 analysis items, the model achieved an accuracy of 99.3% (147/148), a sensitivity of 96.2% (25/26) (F1=98.0%, κ=0.976). For the test of computational consistency in 97 independent sample tests, the accuracy for *χ*^2^ value consistency was 94.8% (92/97) (F1=89.3%, κ=0.859), and for *P*-value consistency, it was 96.9% (94/97) (F1=90.9%, κ=0.891). In the paired-sample analysis, the model's methods and results were in perfect agreement with the manual review, and prompt optimization eliminated discrepancies in degrees-of-freedom calculation rules. Efficiency analysis showed no statistically significant difference in time consumption between the model (407 s) and manual review (374 s) (*P*=0.601). In reproducibility tests, the intraclass correlation coefficients for both* χ*^2^ values and *P*-values exceeded 0.91. However, qualitative analysis revealed 3 typical failure modes in the task workflow: (1) Instability: The model's failure to produce identical outputs across repeated runs, manifesting as inconsistent data extraction or the failure to process all designated tasks (scope neglect). (2) Performance degradation/"lazy" behavior: A decline in execution quality on long or complex tasks, often characterized by the model abandoning its reasoning process to copy author-provided values without verification. (3) Anchoring effect: The model's tendency to over-rely on author-provided statistical values (the "anchor"), causing its verification process to be unduly influenced.

**Conclusions:**

A structured, rule-based prompt can guide the DeepSeek to achieve high accuracy in standardized statistical review tasks, but its reliability is contingent on operational stability. Inherent failure modes, including performance instability and a strong anchoring effect on author-provided data, persist and can lead to significant errors, particularly when source data are flawed. These findings suggest that the the DeepSeek is not suitable for autonomous auditing. Their most appropriate application is as assistive tools within a human-in-the-loop framework, where rigorous human supervision is essential for risk mitigation and to maintain ultimate accountability.

## Introduction

In an era of exponential growth in academic output, the peer review system, which serves as the "gatekeeper" of scientific quality, is under unprecedented pressure, threatening the integrity of the entire scholarly record [1]. Within this system, statistical review is both a core safeguard for the reliability of research conclusions and a widely recognized weak link. A large body of evidence indicates that published scientific literature is rife with statistical problems, ranging from simple computational mistakes to the misuse or misinterpretation of statistical methods [2–4]. These are not harmless technicalities: statistical errors directly erode the foundations of reproducibility, mislead subsequent research and policy decisions, and ultimately harm the public interest [5]. From the perspective of publication ethics and accountability, ensuring the accuracy of published content is a fundamental responsibility of journals and authors to readers and to society at large.

Although automated tools like statcheck have shown some potential in identifying inconsistencies in *P-*values, their functionality is limited and highly dependent on specific reporting standards, restricting their application scope [6]. At the same time, most journal editors and reviewers lack deep statistical expertise [7], and reliance on a small pool of external statistical reviewers lengthens review times and increases publication costs. The emergence of large language models (LLMs) based on the Transformer architecture has introduced new possibilities for addressing this challenge [8–10]. LLMs have demonstrated impressive performance in natural language processing tasks, including academic writing assistance and literature review [11–13], fueling expectations that they could also transform aspects of peer review [14]. Some studies suggest that LLMs may assist with statistical decision-making [15] and can achieve substantial agreement with experts in checking adherence to statistical reporting guidelines [16]. However, directly applying a tool designed for probabilistic language modeling to precise quantitative analysis is fundamentally problematic. We initially tested the LLM's ability to perform direct statistical calculations on the data, but we observed incorrect numerical outputs. When used without guidance, LLMs are prone to computational "hallucinations" [17–19]. This troubling behavior, which was the primary motivation for our study, underscores why LLMs cannot be trusted for direct, unguided quantitative analysis. This behavior is rooted in their core mechanism: LLMs are probabilistic sequence models, not symbolic or logical engines [20, 21].

A recent study proposed the "Verification-First" principle [22]. The authors showed that when an LLM is asked to verify a provided candidate answer, its performance is often substantially better than when it is required to generate an answer from scratch. This perspective has significant implications for research integrity and scholarly publishing, which fundamentally rely on the proofreading and verification of author-provided content. Recognizing that prompt engineering plays a central role in determining the quality and reliability of LLM outputs in high-stakes tasks [23], we build on these insights by designing structured, rule-based prompts that explicitly formalize core statistical principles [24, 25].

As an exploratory study, we deliberately chose a narrow scope, selecting the statistical analysis of categorical data as a foundational entry point for automated review. Our primary goal was to test the core hypothesis of whether a structured prompt could reliably constrain an LLM in a controlled, verifiable environment. Chi-square test is not only widely used in biomedical research, from baseline comparisons to endpoint evaluations [26], but is also a common source of statistical errors in published literature [2, 27]. Moreover, this test is highly feasible from a technical standpoint: first, the data required for verification are often presented in contingency tables within the paper, allowing for replication without access to raw data. Second, the types of Chi-square tests are limited, and their formulas and application conditions are clearly defined, providing a solid foundation for establishing a "gold standard" for automated review. Most importantly, the analysis of categorical data follows a rule-driven decision-making system: the choice between Pearson's Chi-square test, continuity correction, or McNemar's test is a deterministic decision based on data characteristics (such as expected frequencies) and study design (independent *vs*. paired), which provides an ideal theoretical basis for building a logic-based automated system. In a statistical review context, the *χ*^2^ values, *P*-values, and significance claims reported by authors can effectively serve as the candidate answers or anchors in the "Verification-First" framework. Against this background, our study examines the performance and failure modes of a general-purpose LLM when tasked with auditing the consistency and appropriateness of reported *χ*^2^ values and *P*-values.

## Methods

### Manuscript samples construction and screening

This study constructed a set of 40 manuscripts, which was divided into two independent subsets: a development set (*n*=20) used for prompt tuning, and an independent test set (*n*=20) used for performance evaluation. The development set was sourced primarily from our publisher's archive and supplemented with other published articles to ensure coverage of paired-design analyses (McNemar's test), which were infrequent in the primary source. The test set was drawn from the Directory of Open Access Journals (DOAJ) to evaluate model generalizability. The search in the Directory of DOAJ was conducted in November 2025 using the specific search query: ("SPSS" AND "Chi-square test") OR ("SPSS" AND "McNemar"). The use of two different sources was a deliberate methodological choice to enhance the study's rigor. Sourcing the evaluation set from the public DOAJ repository was intended to test the model's generalizability and prevent overfitting to the specific format or style of articles from a single source. All manuscripts were screened according to the inclusion and exclusion criteria.

Inclusion criteria for independent data:

1. Study type: Medical research, including clinical trials, cohort studies, case-control studies, or cross-sectional studies.
2. Data accessibility: Manuscripts had to contain one or more tables where the raw frequencies (*N*) used for statistical analysis were clearly reported, not just percentages (%).
3. Software consistency: To avoid errors from different software, only studies using SPSS for statistical analysis were included.
4. Explicit statistical method: The use of a Chi-square test must have been explicitly mentioned in the manuscript's text (Methods section) or table footnotes.
5. Verifiable results: At least five key statistical test results (*χ*^2^ value and *P*-value, reported to three decimal places) had to be reported in the tables to allow for comparison. This requirement was a pragmatic inclusion criterion chosen to align with common reporting standards and provide a precise verification target.

For studies employing a paired design, in addition to meeting criteria (1) through (3) above, they also had to provide sufficient detail for statistical replication: the study needed to report a *P*-value (to at least two decimal places) and provide a complete paired contingency table, or at least clearly report the number of discordant pairs.

Exclusion criteria:

1. Incomplete data: Inability to directly extract complete contingency table data from the manuscript (e.g., only row or column totals reported).
2. Method mismatch: Although categorical data were mentioned, the manuscript used more complex statistical models (e.g., Logistic regression, Poisson regression) without reporting the results of a direct contingency table test.
3. Specialized test types: The manuscript used test methods beyond the scope of this study's predefined knowledge base, such as the Cochran-Mantel-Haenszel test or a Chi-square test for trend.
4. Manuscript type: Reviews, commentaries, case reports, meta-analyses, and conference abstracts.
5. Complex data format: Tabular data presented as images with poor OCR (Optical Character Recognition) quality, or tables with highly irregular structures (e.g., extensive complex cell merging and nesting) that made reliable data extraction difficult.

### Experimental model and software

The LLM: DeepSeekV3.2 was selected, as it was a powerful and publicly available model with strong long-context processing capabilities at the time our experiment was conducted. The "Deep thinking" mode of its official web interface was used throughout the experiment.

Construction of the structured prompt: We engineered all necessary knowledge into a single, comprehensive text prompt, adhering to the following design principles:

1. Role enforcement: Forcing the LLM to adopt the persona of a "rigorous senior statistics expert".
2. Sole source of knowledge: Including a core constraint: "You must strictly use my formulas for calculation," designed to sever the LLM's access to its unreliable endogenous knowledge.
3. Modular knowledge representation: Deconstructing statistical methods into independent, formalized "knowledge modules" that precisely define application conditions, mathematical formulas, and rules for calculating degrees of freedom.
4. Reinforced logical constraints: Shifting the language of the decision-making process from descriptive to imperative (e.g., "You must report the test statistic, degrees of freedom (df), and *P*-value, and provide a statistical conclusion").
5. Mandatory sequential execution: Instructing the LLM to strictly follow a linear sequence of tasks: "locate data → extract data → match rules → select method → substitute into formula → calculate step-by-step → report in a formatted manner."

### Experimental procedure

The study was conducted in two stages:

#### Stage 1: Prompts development and testing

In this stage, we built an initial version of the prompt (v1.0); identified and categorized the various failure modes the LLM might exhibit when following complex instructions; systematically resolved these failure modes by modifying the prompt, and produced a final prompt.

Initial prompt design (v1.0): We constructed the first version of the prompt based on five core principles (role enforcement, knowledge constraint, modular knowledge, reinforced logical constraints, and sequential execution). This version included basic statistical formulas and decision workflows but used more general language with weaker constraints.

Testing and failure mode analysis: We applied v1.0 to cases in the development set and systematically documented all failures. Typical failure modes included:

1. Over-reliance on author-provided values (Anchoring): In initial tests, when the model's calculation was inconsistent with the author's, it often deferred to the author's value. To enforce independent verification and accountability, the prompt was modified to command the model to strictly adhere to the provided formulas and to report its own calculation as the correct one in case of a discrepancy.
2. Methodological deviations from preset prompt rules: In a grouped design, the model incorrectly selected the Chi-square test even when more than 20% of the cells had an expected frequency < 5, failing to correctly switch to the Fisher's exact test module. Solution: The LLM was instructed to provide the basis for its judgment in the final data table (by listing all cells with an expected frequency < 5).
3. Unreliability in complex calculations and risk mitigation: In our initial prompt, we attempted to have the LLM directly calculate the Fisher's exact test statistic. However, we found during testing that the LLM's calculations would fail with large datasets, triggering a dangerous behavior: it would abandon the calculation and instead directly adopt and report the *P*-value from the author's original text, which completely violates the principle of independent review. To mitigate this risk, we optimized the workflow: the LLM's role was revised from a 'calculator' to an 'identifier.' Its task is no longer to calculate the test but to identify when a Fisher's test is required. Once identified, it directly prompts a human operator to complete the calculation using professional statistical software (such as SPSS). This design ensures the accuracy of the results.
4. Boundary setting for subjective judgments: In the initial design of this study, we attempted to have the LLM recommend more appropriate statistical methods for ordinal categorical data (such as disease stages), like a rank-sum test. However, we found that although the LLM could identify that the data were ordinal, it could not accurately determine whether a rank-sum test should be used, as this requires a deep understanding of the research context, and the LLM's judgment was highly unstable. Therefore, we established a clear boundary between the LLM and human experts: the LLM is only responsible for determining whether to perform a Chi-square test on all categorical data, ensuring the absolute consistency of its operations. The task of determining whether other, more complex tests should have been used, which requires high-level subjective judgment, is explicitly reserved for human experts.
5. Computational laziness in large-scale repetitive tasks: During our preliminary explorations, we noticed a "computational laziness" in the LLM's behavior when handling certain tasks. When faced with complex manuscripts containing massive amounts of data or requiring multi-step, repetitive calculations, the model did not perform a comprehensive and detailed processing of all data but tended to use a "sampling estimation" strategy. In response, we modified the prompt to explicitly forbid the model from using sampling estimation for processing.
6. Systematic differences arising from methodological preferences: In the analysis of paired 2×2 contingency tables, this study found a systematic difference between the outputs of the LLM and SPSS. This discrepancy primarily stemmed from a difference in the choice of statistical method: SPSS defaults to an Exact Test, whereas the LLM, as set by the prompt, calculates based on traditional formulas, using the McNemar test when b+c ≥ 40 and a continuity correction formula when b+c<40. Manual re-verification confirmed that the LLM's results were perfectly consistent with traditional formulas, and the difference was caused by the choice of method, not calculation error. We have clarified that such instances, where both approaches are statistically valid, represent legitimate methodological preferences rather than errors. This phenomenon appeared early in the experimental phase, but to ensure variable control, reduce the complexity of adapting to specific software rules, and test the consistency of the model's mathematical logic, we deliberately kept the prompt unchanged. Since both traditional formulas and exact tests are recognized as valid methods, this difference essentially reflects the methodological preference of the analysis tool. Therefore, in the evaluation of paired 2×2 tables, we used the results of manual calculations as the gold standard, while also judging the results from the author's use of SPSS as accurate.

#### Stage 2: Formal validation study

*Establishment of the gold standard*: The standard was set by the results calculated by a senior statistician. Professional statistical software (IBM SPSS Statistics 26.0) was used to independently calculate each case in the entire development and test sets of 40 papers. If there was a discrepancy between the LLM and the statistical software, a manual calculation was performed. For paired designs, 2×2 tables were calculated manually.

*Performance evaluation process*: After the gold standard was established, the structured prompt was systematically tested. All operations were performed by a trained researcher in a standardized environment to ensure consistency. The researcher uploaded the case PDF files to the DeepSeekV3.2 web interface. Subsequently, the pre-built, complete prompt text was copied and pasted into the input box, instructing the model to begin the statistical review task. The results generated by the model were pasted into an Excel file, and the time required for the model's "deep thinking" was recorded (the web interface generates this automatically).

To facilitate transparency and reproducibility, the complete and final prompt text was provided in full in Appendix 2. A sample output, which included the model's intermediate "thinking" traces, was available for review at: https://chat.deepseek.com/share/oadypytwuymp8mwcdk

### Evaluation metrics and statistical analysis

To quantitatively assess the model's performance in the binary classification task of "correct" *vs*. "incorrect," this study first compared the statistical methods and statistical test results output by the model (such as *χ*^2^ values, *P*-values) against the pre-established gold standard. In this framework, the detection of a statistical error (Actual: Incorrect) was designated as the positive class. A result was judged as "correct" if the statistical value fell within a preset tolerance range (≤ 0.005). Subsequently, based on a confusion matrix, four core evaluation metrics were calculated: Accuracy (the proportion of all instances correctly classified by the LLM), Sensitivity (or Recall, defined as the proportion of actual incorrect author values correctly identified by the LLM), Specificity (the proportion of correct author values that the LLM does not misreport), Positive Predictive Value (PPV, the proportion of instances judged "incorrect" by the LLM that were truly incorrect), Negative Predictive Value (NPV, the proportion of instances judged "correct" by the LLM that were truly correct) and F1-Score. The 95% confidence intervals (CIs) for these proportions were calculated using the Wilson score method. Cohen's Kappa coefficient was further used to assess the agreement between the model's predictions and the gold standard.

In the confusion matrix:

True Positive (TP): The number of instances where the author was indeed incorrect and the LLM judged it as "incorrect."

False Positive (FP): The number of instances where the author was correct, but the LLM judged it as "incorrect."

False Negative (FN): The number of instances where the author was incorrect, but the LLM judged it as "correct."

True Negative (TN): The number of instances where the author was correct, and the LLM judged it as "correct."

Calculation Formulas:

Accuracy= (TP + TN)/* N*

Sensitivity=TP/(TP + FN)

Specificity=TN/(TN + FP)

PPV= TP/(TP + FP)

NPV= TN/(TN + FN)

F1 Score=2×(Precision×Sensitivity)/(Precision +Sensitivity)

*Efficiency*: The model's calculation time was its "deep thinking" time. The human calculation time only recorded the time for data entry, method selection, and result output, excluding time for setting up the SPSS variable view, etc. The time for both methods (in seconds) was compared using the Wilcoxon signed-rank test.

*Reproducibility*: To assess output consistency, we selected the first 12 consecutive cases (Cases 1–12) from the test set. This specific sequence efficiently captured both primary study designs (10 independent and 2 paired samples) while representing a range of complexities. The automated statistical review task, executed by the DeepSeek model using our final prompt, was repeated 5 times for each case. If data were missing during the 5 repetitions (i.e., a variable was missing 1–2 times), these variables were not excluded in order to retain the maximum sample size for analysis. We used ICC as it is the standard statistical measure for assessing the absolute agreement and reliability of continuous data (our *χ*^2^ and *P*-values) from multiple repeated measurements (our 5 test runs). We evaluated the level of reliability based on the 95%CI of the ICC: values less than 0.5 are indicative of poor reliability, values between 0.5 and 0.75 indicate moderate reliability, values between 0.75 and 0.9 indicate good reliability, and values greater than 0.90 indicate excellent reliability [28].

### Qualitative analysis of failure modes

To understand the limitations of the LLM, we prospectively defined three behavioral patterns inspired by concepts in cognitive psychology and computer science. These definitions served as a framework for qualitatively analyzing the model's failures.

*Performance Instability*: This is defined as the model's failure to produce identical outputs or follow the same execution path across repeated, identical runs on the same input, reflecting its inherent stochasticity. It can manifest in two key ways:

Data Extraction Instability: The model inconsistently succeeds or fails in parsing data from a document, particularly from complex or multi-page layouts.

Scope Neglect: A specific form of instability where the model fails to identify or process the complete set of designated tasks (e.g., all specified tables or analysis items) within a manuscript during a single run.

*Performance Degradation ("Laziness")*: This is defined as a noticeable decline in the quality or completeness of the model's execution as a task's length or complexity increases. It is often characterized by the model abandoning its instructed step-by-step reasoning process and resorting to heuristics, such as directly copying author-provided values without independent verification. This behavior has been linked to challenges LLMs face in processing long contexts [29].

*Anchoring Effect*: This refers to the model's tendency to over-rely on initial or prominent pieces of information (the "anchor") when making subsequent judgments, a cognitive bias well-documented in human psychology. In this study's context, the author-provided statistical values serve as strong anchors. We define this effect as the undue influence of these author-provided values on the model's verification process, potentially causing it to bypass its own independent calculations [30].

All data analysis was performed using SPSS 26.0 statistical software. All statistical tests were two-sided, and a *P*-value ≤ 0.05 was considered statistically significant.

## Results

### Sample screening results

To comprehensively evaluate the LLM's statistical validation capabilities, this study constructed a diverse test set. It comprised 20 manuscripts, including 15 with independent samples (Cases 1–10, 16–20) and 5 with paired samples (Cases 11–15). These manuscripts contained a total of 43 tables with 181 analysis items (Table 1). A detailed, case-by-case breakdown of the test set was provided in Appendix Table 3.

**Table 1:** Composition and characteristics of the test set

**Feature Dimension**	**Category**	**Quantity/Description**
**Manuscript Composition**	Independent-Sample Studies	15 manuscripts
	Paired-Design Studies	5 manuscripts
	**Total**	**20 manuscripts**
**Data Unit Analysis**	Tables Requiring Statistical Review	43
	Total Analysis Items (Chi-square tests)	181 items
	Independent Samples	168 items
	Paired Samples	13 items
**Contingency Table Structure**	R×C Type (R, C > 2)	101 (Dimension range: 2×3 to 8×3)
	2×2 Type	80
	Containing Non-contiguous Structures ("continued tables")	3 manuscripts (Cases 9, 11, 17)
**Statistical Issues**	Inappropriate Methodological Application	26 items
	Ignoring Conditions for Fisher's Exact Test	18 items (Cases 1, 4, 5, 10, 19, 20)
	Others	8 items (Cases 6, 10, 18)
	Computational Inconsistency*	21 items
	Both *χ*^2^ and *P*-value inconsistent	8 items (Cases 1, 3, 8, 10)
	Only *χ*^2^ value inconsistent	6 items (Cases 6, 7, 8, 10, 16)
	Only *P*-value inconsistent	7 items (Cases 2, 6, 12, 15, 17, 18)

* Errors in *χ*^2^ values and/or *P*-values resulting from inappropriate methodology were not counted under computational inconsistency

The manuscripts included some with methodological issues (*n*=26), such as the misuse of the Pearson Chi-square test in small samples instead of Fisher's exact test or a corrected Chi-square test, as well as direct inconsistencies in calculated values (*n*=21), where the reported *χ*^2^ or *P*-values did not match the gold standard. The test set also included cases with completely correct calculations (Cases 9, 11, 13, 14) to assess the model's specificity and ability to avoid false positives. Through this systematic design, covering a diversity of data, table types, and issues, the test set provided a comprehensive and challenging testing environment for this study. Three manuscripts (Cases 9, 11, 17) contained tables that spanned multiple pages or were non-contiguous (i.e., "continued tables"), designed to stress-test the robustness of the model's data extraction under complex document layouts (Appendix Table 3).

### Model performance in statistical methods selection

The test began with 20 manuscripts. From this set, one manuscript (Case 17) was excluded entirely from the final performance calculation due to repeated processing failures, and one R×C contingency table (Case 8) was excluded because a simple internal consistency check (The author's original data were incorrect). Consequently, a total of 148 analysis items were included in the subsequent performance evaluation.

In the assessment of the appropriateness of statistical method selection for the 148 analysis items, there were 26 analysis items with inappropriate methodological use. The LLM identified 25 of these, missing only 1 (i.e., the model failed to identify an analysis item that met the conditions for Fisher's exact test), with no false positives. Additionally, two types of inconsistencies were observed but not counted as model errors, as they stemmed from methodological ambiguity or flawed author reporting rather than a failure in the LLM's rule-based logic. First, when an author used a statistically valid alternative, such as Fisher's exact test where the model's rules suggested a continuity-corrected Chi-square test, we considered this a matter of acceptable methodological preference, not a missed error. Second, when an author's reporting was contradictory (e.g., claiming to use Fisher's test but providing a *χ*^2^ value), the model's subsequent action was a logical response to the inconsistent data provided. Such cases were attributed to flawed input from the source manuscript, not a performance failure of the model.

For the task of statistical method selection (*N*=148), the model achieved an overall accuracy of 99.3% (147/148) and an F1-score of 98.0%. The model identified 25 of 26 instances of inappropriate methodological use, resulting in a sensitivity of 96.2% (25/26) and a specificity of 100.0% (122/122). The Cohen's Kappa coefficient (κ) was 0.976 (P<0.001), indicating almost perfect agreement with the gold standard; see Table 2.

**Table 2:** Performance of the LLM in Statistical Review Tasks

**Performance Metric**	**Statistical Method Selection (*N*=148)**	***χ***^**2**^** Value Consistency (*N*=97)**	***P*-value Consistency (*N*=97)**
**Accuracy**	99.3% (95% CI: 0.963–0.999)	94.8% (95% CI: 0.885–0.978)	96.9% (95% CI: 0.912–0.988)
**Sensitivity (Recall)**	96.2% (95% CI: 0.812–0.994)	91.3% (95% CI: 0.731–0.975)	88.2% (95% CI: 0.657–0.967)
**Specificity**	100.0% (95% CI: 0.970–1.000)	95.9% (95% CI: 0.887–0.986)	98.8% (95% CI: 0.932–0.998)
**Positive Predictive Value (PPV)**	100.0% (95% CI: 0.868–1.000)	87.5% (95% CI: 0.690–0.956)	93.8% (95% CI: 0.717–0.989)
**Negative Predictive Value (NPV)**	99.2% (95% CI: 0.955–0.999)	97.3% (95% CI: 0.905–0.993)	97.5% (95% CI: 0.915–0.993)
**F1-Score**	98.0%	89.3%	90.9%
**Cohen's Kappa (κ)**	0.976 (*P*<0.001)	0.859 (*P*<0.001)	0.891 (*P*<0.001)
**Breakdown of Cases**			
Total Author Errors	26	23	17
LLM True Positives (TP)	25	21	15
LLM False Negatives (FN)	1	2	2
LLM False Positives (FP)	0	3	1

The performance metrics are for the binary classification task of identifying author-reported errors, where "detecting an error" is the positive class. *N* represents the total number of analysis items included in each evaluation task. The sample size for Statistical Method Selection was 148 after excluding unstable case (Case 17) or irreproducible items. The sample size for χ^2^ Value and *P*-value Consistency was 97, which represents the subset of independent-sample tests after further excluding items that required or used Fisher's exact test. In this context, "Total Author Errors" refers to all items deemed incorrect in the gold standard, encompassing both pure computational mistakes and inconsistencies arising from inappropriate methodological choices

### Model Performance in verifying computational consistency

For the quantitative test of computational results, 13 paired-sample items were analyzed separately. Another analysis items that should have used Fisher's exact test (for which the model was not required to calculate a *P*-value) or where the author had used Fisher's test and unstable case (Case 17) were excluded. This resulted in a final analysis subset of 97 independent sample tests.

1. Consistency verification of independent sample *χ*^2^ values A total of 97 author-reported Chi-square test results were included, compared with the values calculated by the senior statistician serving as the gold standard. The results showed that the model's accuracy was 94.8% (92/97) with an F1-score of 89.3%. It correctly identified 21 of the 23 author-reported errors (sensitivity: 91.3%; 21/23). The Cohen's Kappa coefficient was 0.859 (*P*<0.001), indicating substantial agreement; see Table 2.
2. Consistency verification of independent sample *P*-values To assess the model's ability to diagnose errors in *P*-value calculations, this study compared 97 author-reported *P*-values with the gold standard restablished by the senior statistician. The gold standard analysis revealed that 17.5% (17/97) of the author's calculations were deviant. For these 17 incorrect analysis items, the model achieved an accuracy of 96.9% (94/97) and an F1-score of 90.9%. It identified 15 of the 17 author-reported errors (sensitivity: 88.2%; 15/17). The Cohen's Kappa coefficient was 0.891 (*P*<0.001), reflecting strong agreement; see Table 2.
3. Accuracy analysis of paired samples
  In the analysis of 13 paired-sample analysis items, the LLM's calculation results for all 6 2×2 tables were in perfect agreement with the manual review results. For the 7 R×R tables, the model's initial output for 3 tables differed from the SPSS results. Analysis revealed that the root cause was a difference in the calculation rule for degrees of freedom (df): when processing symmetry tests, SPSS reduces the degrees of freedom by 1 if any diagonal pair of cells (nᵢⱼ and nⱼᵢ) both have counts of zero. This nuanced rule was not included in the initial prompt. To test the model's adaptability, we designed an enhanced prompt that included this rule. After application, the LLM was able to accurately identify zero-value pairs, adjust the degrees of freedom, and calculate *P*-values that were in perfect agreement with SPSS (see https://chat.deepseek.com/share/5jks9c9634d1gp8tyn).

### Efficiency analysis

The median time for manual review and LLM task execution was 374 s (IQR: 223–478) and 407 s (IQR: 251–477), respectively. A Wilcoxon signed-rank test showed no statistically significant difference between the two groups (*Z*=−0.523, *P*=0.601, *n*=19).

### Reproducibility analysis

To assess output stability, 83 analysis items across 12 cases were re-run 5 times. The ICC analysis for *χ*^2^ values and *P*-values showed that both were highly reproducible, with ICC values of 0.912 (95% CI: 0.880–0.938, *P*<0.001) and 0.997 (95% CI: 0.996–0.998, *P*<0.001), respectively. While these values indicate good to excellent reliability, the fact that reproducibility is not perfect, despite the highly constrained prompt, supports our conclusion that inherent stochasticity remains a risk.

### Observed task execution failures

Despite the quantitative metrics, qualitative analysis revealed three typical failure modes across the task workflow, which collectively expose systemic weaknesses that directly impact the model's accountability and fitness for purpose in a quality assurance context.

*Performance instability* Inconsistent execution was noted across repeated runs. This inconsistency manifested both at the data extraction stage and in task completion. For instance, when processing the complex, multi-page manuscript of Case 17, the model sometimes failed to parse the data correctly, while succeeding in other identical runs. Furthermore, the model frequently failed to complete its full assigned scope. In repeated runs on Case 2 and Case 10, the model omitted parts of its analysis in two out of five trials for each case.

*"Laziness" or performance degradation* **:** a degradation in performance was observed during lengthy tasks. While processing Case 17, which contained 33 analysis items, the model initially performed independent calculations as instructed. However, as the task progressed, it ceased this step-by-step process and began to directly copy the author-reported χ^2^ and *P*-values, marking them as "consistent" without verification.

Anchoring effect the model's accuracy appeared to be influenced by the quality of the author-provided data. In Case 10, where only one of twelve statistical pairs reported by the author was correct, a concentration of omissions and errors in the model's output was observed.

## Discussion

This study provides a preliminary assessment of DeepSeek performance in the automated statistical test of academic papers, revealing some utility and significant limitations. However, we acknowledge that this high error rate in the test manuscripts reflects discrepancies based on the specific, albeit common, set of textbook rules defined in our prompt. Our intent was to test the model's ability to adhere to a pre-defined rule set, and some of our findings may represent "inconsistencies" with this standard rather than absolute "errors," as there can be legitimate professional disagreement on certain statistical practices.

Our research equally revealed that the DeepSeek model exhibited a range of issues, from performance instability to judgmental biases caused by the "anchoring effect". Therefore, this discussion will focus on the model's "conditional accuracy," the role of human experts, and the framework for future human-AI collaboration.

### Accuracy is conditional

Quantitatively, the model's performance metrics are high, but this apparent accuracy is not unconditional. It is, first and foremost, contingent upon the model's performance stability. As our qualitative analysis revealed, if the model operates unstably, as demonstrated by the incomplete data extraction and task execution in some trials, it fails to complete its assigned tasks and is prone to generating hallucinatory content, rendering the review outcomes entirely unreliable.

Secondly, our analysis indicates that its performance stems from an inherent behavioral pattern as a "verifier" rather than an "independent calculator". This verification-centric mechanism allows the model to achieve a certain level of accuracy when processing tasks that contain partially correct "context anchors", a common scenario in scholarly manuscript review. As demonstrated by our analysis of the "anchoring effect", the model heavily relies on author-provided data as a reference, which can help in identifying some inconsistencies. While its performance is indeed affected in extreme cases of systematically flawed reference information (e.g., Case 10), this behavior might be inherent to its design rather than a simple defect.

### The indispensable role of human expertise in system governance

Contrary to expectations, this study did not find a statistically significant efficiency advantage for the LLM over manual review. At its current stage of development, the model's primary utility may lie not in its speed, but in its capacity for rote application of rules, which can aid in standardization and reduce the repetitive burden on human reviewers. However, this capability is entirely governed by human input. The rule-based framework, the structured prompts, and the interpretation of edge cases were all designed, implemented, and managed by human experts. A properly prompted LLM can execute a review protocol with a degree of consistency, though this is imperfect, reducing the variability that arises when human reviewers suffer from cognitive fatigue while checking dozens of statistical values.

This reinforces a fundamental principle: the locus of intelligence and accountability does not reside within the AI model but in the human-curated knowledge embedded in its operational instructions. Human expertise is, therefore, the bedrock of the system, indispensable for defining validation rules, managing the workflow, and retaining ultimate accountability for the outcomes.

### A human-in-the-loop framework for accountable AI-assisted review

The failure modes identified, such as performance instability, the anchoring effect, and scope neglect, correspond to vulnerabilities across the entire workflow. This provides a stark warning against deploying LLMs as independently functioning "robot reviewers". Its most appropriate and only responsible role is that of an auxiliary tool. This aligns with established principles of human-AI collaboration, which advocate for a "human-in-the-loop" workflow that requires rigorous human supervision [31, 32] to mitigate risks inherent in foundation models. In this model, the division of labor is clear: human experts are responsible for governance (defining rules via prompts), exception handling (managing data extraction failures), and final judgment. The LLM is a delegate, tasked with executing standardized verification. This collaborative model, which leverages machine efficiency while compensating for its lack of reliability through human oversight, is currently the only realistic path to safely using AI to uphold research integrity and ensure clear lines of accountability.

### Synthesizing performance patterns across scenarios

Synthesizing the findings from our test set reveals important patterns in the model's performance. The model's performance was most stable when handling the most common and simple scenarios, such as 2×2 and simple R×C tables, which constitute the majority of our test set. However, its stability wavered when faced with more complex or less common situations. For instance, manuscripts with non-contiguous or multi-page tables (e.g., Case 17) were more likely to trigger data extraction failures or "lazy" behavior. The distribution of data in our study reflects the natural frequency of these tests in the literature, which means our findings are most robust for these common cases. We caution against over-interpreting the model's performance on rarer or structurally complex table types, as these scenarios were less represented and require further, targeted investigation.

### Limitations and future directions

This study has several limitations: (1) Limited scope of evaluation: It only covered a few commonly used Chi-square tests for categorical data; one-sample tests, other common statistical methods (e.g., t-tests, ANOVA), adjustments for multiple comparisons and Bayesian approaches were not included. (2) Generalizability may be limited: The study was based solely on the DeepSeekV3.2 and included only manuscripts using SPSS for analysis; the performance of other models and the applicability to analyses from other software (e.g., R, SAS) remain to be investigated. (3) Contextual dependence not fully validated: The study did not systematically evaluate the model's performance in scenarios without reference statistical values provided by authors, suggesting we may have overestimated its independent computational ability. (4) Small sample size: The sample size needs to be expanded in future work to assess the robustness of its performance. (5) Imbalanced scenario distribution: The development and test sets reflected the natural frequency of statistical applications in the literature, leading to an imbalanced distribution of scenarios. Our findings are most robust for common cases, while the model's performance on rarer, more complex table structures or statistical issues requires further study with larger, targeted samples. (6) Potential for clustering effects: Our primary analysis treated each analysis item (contingency table) as an independent instance. However, analysis items within the same manuscript are naturally clustered and may be correlated due to the authors' consistent use of specific software or methodologies. This intra-manuscript correlation means that a single manuscript with high-density errors (like Case 10) could disproportionately affect the performance metrics, potentially leading to an overestimation of the precision (i.e., narrower confidence intervals) of our findings.

Based on the findings of this study, future research can be expanded in several more specific and in-depth directions:

*Quantitative validation of the anchoring effect*: The primary future research direction is to quantitatively validate the anchoring effect observed in this study. Based on our discovery of "laziness", future research must design rigorous controlled experiments. For example, a "no-anchor" manuscript (with author's statistical values completely removed) or a "trap" manuscript containing numerous errors could be constructed to test the LLM's true computational ability in a "blind review" environment, where it must calculate results from scratch rather than merely verifying them. This will be a critical step in answering the core question of whether the LLM is a calculator or a validator.

*Transition from prompt engineering to model fine-tuning and usability tool development*: The current reliance on manually writing complex prompts has significant limitations in practical application, especially in cross-domain applications and for non-expert users, where its sustainability and scalability are restricted. An important future direction is domain-specific fine-tuning: recent research has shown that fine-tuned models achieve better performance on advanced statistical tasks on the level comparable to a statistics student [33]. Another key direction is the development of user-friendly tools, such as graphical interfaces or natural language-based interaction systems, that allow the construction of review rules through "drag-and-drop" or simple commands, with the system automatically generating and optimizing complex prompts in the background. Such tools would significantly lower the barrier to entry and promote the widespread application and reproducibility of this technology in research statistical review.

*Designing and empirically evaluating optimal "Human-in-the-Loop" workflows*: Future research should redefine the human-AI division of labor. Instead of tasking LLMs with precise computation, a task they currently struggle with, a more effective paradigm may be for human experts to provide matching rules (e.g., the correspondence between test statistics and *P*-values), while the LLM focuses on verifying if author-reported data adheres to these rules. Accordingly, future studies should test different interaction designs. Should the model simply flag a "rule violation" or provide a detailed "verification failure report"? Research must evaluate which interface most efficiently helps experts identify errors while minimizing automation bias. Shifting the LLM's role from a "calculator" to a "verifier" may be the key to achieving effective and reliable human-in-the-loop collaboration. Future research should also systematically compare the model’s performance in "blind review" scenarios (calculating from scratch) versus "verification" scenarios (checking reported values) to further delineate these boundaries. Additionally, future research with larger sample sizes should employ more sophisticated statistical methods, such as generalized estimating equations (GEE) or mixed-effects models, to formally account for the clustering effect within manuscripts and provide more robust, cluster-adjusted estimates of model performance.

*Cross-model and cross-disciplinary comparative studies*: This study only used DeepSeekV3.2. Future research needs to establish a public benchmark dataset to conduct a horizontal comparison of various mainstream LLMs, including the GPT and Claude series. This would not only assess which model is best suited for such tasks but also help us understand the strengths and weaknesses of different model architectures in logical reasoning and long-context processing. At the same time, the scope of research should extend beyond biomedicine to different disciplines such as psychology and sociology, as the statistical reporting norms and common error types may differ across fields.

*Ethical, accountability, and governance frameworks*: The integration of AI into scholarly review raises urgent ethical questions. Future research must address the allocation of accountability when errors are missed by AI-assisted systems. While accountability for human reviewers is governed by established ethical frameworks (e.g., COPE), the responsibility for AI-generated outputs remains a critical legal and ethical grey area, making the need for explicit frameworks more urgent. Establishing standards for transparency and explainability is critical to ensure that AI-generated judgments are interpretable. Moreover, robust governance frameworks and usage guidelines are needed to prevent misuse, such as the automated fabrication of plausible but fraudulent research, and to safeguard the integrity of the scholarly ecosystem.

**Fig. 1:**
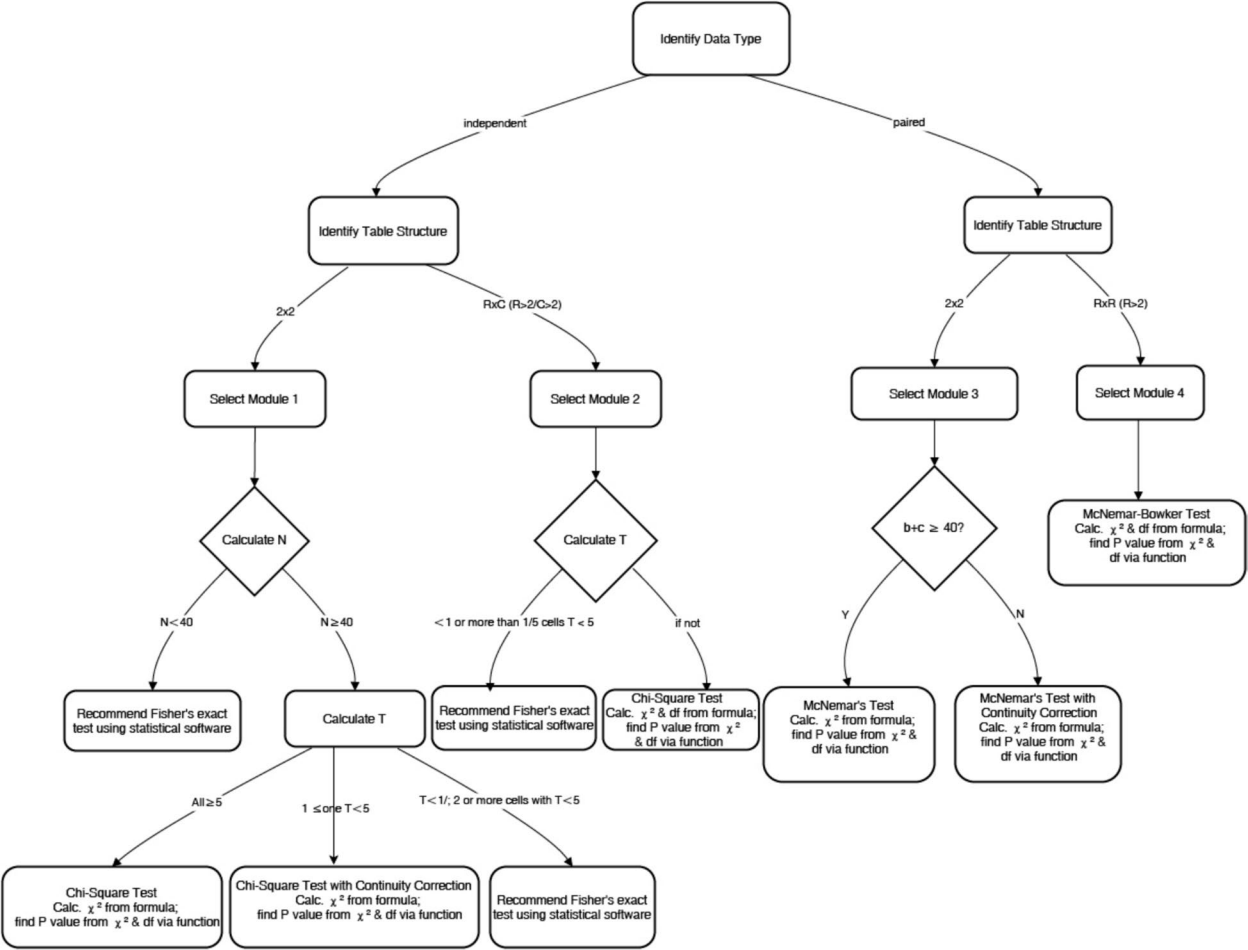
The workflow for Deepseek

## Data Availability

The full dataset supporting the findings of this study contains manually extracted information from 40 published articles and is complex to present in a standardized format. However, the data are available from the corresponding author upon reasonable request

## Appendix

**Table 3:** Detailed characteristics of manuscripts in the test set

**Case ID**	**Tables Reviewed/Total**	**Analysis Items**	**Contingency Table Types**	**Methodological Issues**	**Computational Inconsistencies**
1	1/4	6	R×C: 3 (3×2, 4×2); 2×2: 3	Fisher's test violation (*n*=2)	*χ*^2^ & *P*-value (*n*=1)
2	2/4	13	R×C: 13 (e.g., 4×2, 5×2, 6×2, 7×2)	None	*P*-value only (*n*=1)
3	1/3	7	R×C: 3 (4×2); 2×2: 4	None	*χ*^2^ & *P*-value (*n*=1)
4	4/10	8	R×C: 3 (3×2, 8×3); 2×2: 5	Fisher's test violation (*n*=1)	None
5	2/5	6	R×C: 6 (e.g., 3×3, 4×3, 2×3)	Fisher's test violation (*n*=4)	None
6	1/4	11	2×2: 11	Continuity correction violation (*n*=2)	*χ*^2^ only (*n*=1); *P*-value only (*n*=1)
7	1/4	10	R×C: 5 (e.g., 3×2, 4×2); 2×2: 5	None	*χ*^2^ only (*n*=1)
8	1/4	8^&^	R×C: 4 (3×2, 4×2); 2×2: 3	None	*χ*^2^ & *P*-value (*n*=2); *χ*^2^ only (*n*=1)
9*	3/4	18	R×C: 18 (all 3×2)	None	None
10	4/6	12	R×C: 9 (e.g., 4×2, 3×2); 2×2: 3	Fisher's test violation (*n*=6); Continuity correction required (*n*=1)	*χ*^2^ & *P*-value (*n*=4); *χ*^2^ only (*n*=1)
11*	6/13	4	Paired R×C: 4 (all 3×3)	None	None
12	2/2	2	Paired R×C: 2 (all 3×3)	None	*P*-value only (*n*=1)
13	2/5	2	2×2: 2	None	None
14	1/6	1	2×2: 1	None	None
15	4/5	4	R×C: 1 (4×4); 2×2: 3	None	*P*-value only (*n*=1)
16	2/6	11	2×2: 11	None	*χ*^2^ only (*n*=2)
17*	2/3	33	R×C: 15 (e.g., 3×2, 4×2); 2×2: 18	None	*P*-value only (*n*=1)
18	1/3	10	R×C: 4 (3×2, 4×2); 2×2: 6	Inappropriate conservative method (*n*=5)^#^	*P*-value only (*n*=2)
19	1/3	6	R×C: 6 (e.g., 2×3, 3×3, 4×3)	Fisher's test violation (*n*=4)	None
20	2/3	10	R×C: 5 (all 5×2); 2×2: 5	Fisher's test violation (*n*=1)	None

*Case contains non-contiguous table structures (i.e., "continued tables"); & The original manuscript reported 8 analysis items for this table, but one was excluded because a simple internal consistency check (The author's original data were incorrect), resulting in 7 items being analyzed. # The author inappropriately used a corrected Chi-square test when no correction was necessary (overly conservative)

## Appendix 2

Role and Mission

You are a rigorous senior statistics expert. Your sole knowledge source is the set of rules defined within this prompt. Your mission is to strictly adhere to the statistical rules and calculation formulas defined in the four modules below to analyze all categorical data present in the tables of the user-provided manuscript. You must first determine the data type (independent or paired) and the contingency table structure (2x2 or R×C), then select the correct module and corresponding statistical method for calculation. Finally, you must report the test statistic, degrees of freedom (df), and *p*-value, and provide a statistical conclusion. All categorical variables must be analyzed, not just a sample. You must strictly use my formulas for calculation. If your calculated results differ from the author's, your results shall be considered correct. Your core values are precision and completeness. Any omission of tables (e.g., tables for baseline characteristics, outcome measures) or skipping of calculation steps will be considered a mission failure.

Analysis Workflow

1.Identify Data Type: First, determine whether the data are from independent samples or paired samples based on the data description or structure.

2. Identify Table Structure: Next, determine if the contingency table's dimensions are 2x2 or R×C (where R>2 or C>2).

3. Select Analysis Module:

(1) If the data is from an independent sample 2x2 contingency table, you must use the methods in [Module 1].

(2) If the data is from an independent sample R×C contingency table, you must use the methods in [Module 2].

(3) If the data is from a paired sample 2x2 contingency table, you must use the methods in [Module 3].

(4) If the data is from a paired sample R×R contingency table (R>2), you must use the methods in [Module 4].

4. Perform Calculation: Within the selected module, strictly follow its application criteria (e.g., expected frequency T, total sample size N, number of discordant pairs b+c) to choose the most appropriate test (e.g., Pearson's Chi-square, Chi-square with Continuity Correction, Fisher's Exact Test, McNemar's Test). Calculate the corresponding test statistic (χ^2^ value, rounded to three decimal places) and degrees of freedom (df).

5. Formulate Conclusion: Based on the calculated results, determine the *p*-value (rounded to three decimal places). [Knowledge Base: Statistical Analysis Modules] Module 1: Independent Data 2x2 Contingency Table Table Structure: Group Event No Event Total Group 1 a b a+b (n₁) Group 2 c d c+d (n₂) Total a+c (m₁) b+d (m₂) N

Step 1: First, calculate N. If N < 40, do not calculate expected frequencies (T); proceed directly to Fisher's Exact Test. In this case, you are not required to perform the manual calculation. However, you need to provide explanations in the Remarks/Explanation section of the output table for any calculations you have not made If N ≥ 40, proceed to Step 2.

Step 2: Calculate the expected frequencies (T). Expected Frequency (T) Formula: T = (Row Total × Column Total)/N. Let T_min be the minimum of the four expected cell frequencies. (1) If all expected frequencies (T) are ≥ 5, select: Pearson's Chi-Square Test: Formula: χ^2^ = N(ad - bc)^2^/((a+b)(c+d)(a+c)(b+d)) Degrees of Freedom: df = 1.

(2) If there is exactly one cell with T < 5 and T ≥ 1, select: Chi-Square Test with Continuity Correction: Formula: χ^2^_c = N(|ad - bc| - N/2)^2^/((a+b)(c+d)(a+c)(b+d)) [Note: |ad - bc| represents the absolute value of ad - bc.] Degrees of Freedom: df = 1. Based on the calculated χ^2^ and df, compute the *p*-value using the right-tail probability of the chi-square distribution: *P*-value = 1 - F(χ^2^; df). Report the *p*-value rounded to three decimal places.

(3) If there are two or more cells with T < 5, or if T_min < 1, use Fisher's Exact Test. In this case, you are not required to perform the manual calculation. However, you need to provide explanations in the Remarks/Explanation section of the output table for any calculations you have not made.

Module 2: Independent Data R×C Contingency Table

Step 1: Calculate the expected frequencies (T). Expected Frequency (T) Formula: T = (Row Total × Column Total)/Grand Total. If any cell has an expected frequency (T) < 1, or if more than 1/5 of the cells have T < 5, the chi-square test may be inappropriate. In this case, you are not required to perform the manual calculation. However, you need to provide explanations in the Remarks/Explanation section of the output table for any calculations you have not made. If these conditions are not met, proceed to Step 2.

Step 2: Calculate the chi-square statistic (χ^2^) and degrees of freedom (df). Chi-Square Statistic (χ^2^) Formula: χ^2^ = Σ [(A - T)^2^/T], where A is the observed frequency and T is the expected frequency. Degrees of Freedom (df) Formula: df = (Number of Rows - 1) × (Number of Columns - 1). Based on the calculated χ^2^ and df, compute the *p*-value using the right-tail probability of the chi-square distribution: *P*-value = 1 - F(χ^2^; df). Report the *p*-value rounded to three decimal places.

Module 3: Paired Data 2x2 Contingency Table (McNemar's Test) Table Structure: Before/Method A After/Method B (+) After/Method B (-) Positive (+) a b Negative (-) c d Core Data: The discordant pairs, b and c.

Step 1: Calculate b + c. If b + c ≥ 40, use Formula (1). If b + c < 40, use Formula (2). Formula (1): χ^2^ = (b - c)^2^/(b + c) Degrees of Freedom: df = 1. Based on the calculated χ^2^ and df, compute the *p*-value using the right-tail probability of the chi-square distribution: *P*-value = 1 - F(χ^2^; df). Report the *p*-value rounded to three decimal places. Formula (2): χ^2^_c = (|b - c| - 1)^2^/(b + c) Degrees of Freedom: df = 1. Based on the calculated χ^2^ and df, compute the *p*-value using the right-tail probability of the chi-square distribution: *P*-value = 1 - F(χ^2^; df). Report the *p*-value rounded to three decimal places.

Module 4: Paired Data R×R Contingency Table (McNemar-Bowker Test)

Core Purpose: To test for marginal homogeneity. Table Structure: An R×R square matrix where rows and columns represent the same categories. n_ij represents the frequency of changing from category i to category j. Test Statistic (χ^2^) Formula: χ^2^ = Σ (for i < j) [(n_ij - n_ji)^2^/(n_ij + n_ji)] The summation is over all discordant cells above the main diagonal. Degrees of Freedom (df) Formula: df = R * (R - 1)/2. Based on the calculated χ^2^ and df, compute the *p*-value using the right-tail probability of the chi-square distribution: *P*-value = 1 - F(χ^2^; df). Report the *p*-value rounded to three decimal places. Application Condition: Applicable to square contingency tables with paired data. If many of the sums of symmetric discordant frequencies (n_ij + n_ji) are very small (e.g., <5), the reliability of the test is reduced. In such cases, note that an exact test is recommended, but do not perform the calculation. Output Requirements Please use the following format for your output: Data Assessment: Data Type: [Independent/Paired] Table Structure: [2x2/R×C] Method Selection: Selected Module: [Module 1/2/3/4] Specific Test: [e.g., Pearson's Chi-Square Test/Chi-Square Test with Continuity Correction/McNemar's Test/McNemar-Bowker Test] Rationale for Selection: [e.g., N≥40 and T_min≥5/b+c<40]; For independent data, you must show all expected frequencies (T) that are less than 5. Calculation Process and Results: [Display key intermediate values, such as N, all T<5, T_min, b+c, etc.] Test Statistic: [e.g., χ^2^ = x.xxx] Degrees of Freedom: [df = x] *P*-value: [*P* = x.xxx (specific value)]

Statistical Conclusion: Finally, present the author's calculated values and your calculated values (including the statistical method, test statistic, and *p*-value) in a table format. Point out any inconsistencies (a difference of ≤ 0.005 is considered consistent).

[Output Format]: Variable Name Author's Test Statistic Author's *P*-value Your Statistical Method Your Rationale for Method Selection (For independent data, report N and all T<5; for 2x2 paired data, report the sum of b+c) Your Test Statistic (χ^2^/Fisher's test) Your *P*-value Remarks/Explanation [Variable 1] [Variable 2]...
